# Detection of Airway Remodeling in Asthma Using Bronchoscopic Optical Coherence Tomography

**DOI:** 10.1016/j.chpulm.2025.100143

**Published:** 2025-02-24

**Authors:** Pieta C. Wijsman, Lisa H. van Smoorenburg, Richard M. van den Elzen, Annika W.M. Goorsenberg, Julia N.S. d’Hooghe, Orestes A. Carpaij, Martijn C. Nawijn, Paul R. Bloemen, Inge A.H. van den Berk, Craig J. Galban, Alex J. Bell, Oliver Weinheimer, Daniel M. de Bruin, Jouke T. Annema, Maarten van den Berge, Peter I. Bonta

**Affiliations:** a Department of Pulmonary Medicine, Amsterdam UMC, Academic Medical Center, University of Amsterdam, Amsterdam, The Netherlands; b Department of Biomedical Engineering and Physics, Amsterdam UMC, Academic Medical Center, University of Amsterdam, Amsterdam, The Netherlands; c Department of Radiology and Nuclear Medicine, Amsterdam UMC, Academic Medical Center, University of Amsterdam, Amsterdam, The Netherlands; d Department of Pulmonary Diseases, University Medical Center Groningen, University of Groningen, Groningen, The Netherlands; e Department of Pathology and Medical Biology, University Medical Center Groningen, University of Groningen, Groningen, The Netherlands; f Groningen Research Institute for Asthma and COPD, Groningen, The Netherlands; g Department of Radiology, Michigan Medicine, University of Michigan, Ann Arbor, MI; h Department of Diagnostic and Interventional Radiology, University of Heidelberg, Heidelberg, Germany; i Translational Lung Research Center Heidelberg, University of Heidelberg, Heidelberg, Germany; j German Center for Lung Research, Heidelberg, Germany

**Keywords:** airway remodeling, asthma, extracellular matrix, high-resolution CT, optical coherence tomography

## Abstract

**Background:**

Airway remodeling is an asthma disease hallmark that relates to asthma severity and progression. We investigated airway wall remodeling using bronchoscopic optical coherence tomography (OCT) to assess airway wall composition reflecting its extracellular matrix components and *high-resolution* CT (HRCT) imaging to assess airway wall thickness (AWT).

**Research Question:**

Can OCT and HRCT imaging be used to detect differences in airway remodeling among healthy control participants, patients with mild to moderate asthma, and patients with severe asthma, and how does remodeling correlate with clinical disease severity and other parameters?

**Study Design and Methods:**

The study population included 16 healthy control participants, 15 patients with mild to moderate asthma, and 18 patients with severe asthma. All participants were characterized extensively clinically, and both OCT and HRCT imaging were performed.

**Results:**

OCT imaging high-intensity scattering area was increased in patients with severe asthma in medium airways compared with patients with mild to moderate asthma and healthy control participants. HRCT imaging-derived AWT was significantly higher in patients with asthma when compared with that of healthy control participants, but did not differentiate between levels of asthma severity. Overall in patients with asthma, a higher HRCT imaging AWT and OCT imaging high-intensity scattering area were associated with poor asthma control. Additionally, a thicker airway wall was associated with more severe airflow obstruction and higher blood eosinophil and neutrophil counts, whereas a larger high-intensity scattering area was associated with a lower number of blood eosinophils.

**Interpretation:**

OCT and HRCT imaging provide different and additional information on airway wall remodeling in asthma. Bronchoscopic OCT imaging high-intensity area increases with asthma severity and correlates with poor asthma control, which emphasizes the potential of OCT imaging for assessing disease severity and therapeutic responses in patients with asthma.

**Trial Registry:**

ClinicalTrials.gov; Nos.: NCT03141814 and NCT02225392; URL: www.clinicaltrials.gov

**Take-Home Points**

**Study Question:** Can optical coherence tomography (OCT) and high-resolution CT (HRCT) imaging be used to detect differences in airway remodeling among healthy control participants, patients with mild to moderate asthma, and patients with severe asthma, and how does remodeling correlate with clinical disease severity and other parameters?

**Results:** Increased high-intensity scattering areas—reflecting extracellular matrix components and detected by OCT imaging—and an increased airway wall thickness detected by HRCT imaging both correlate with poor asthma control, but provide different insights into airway remodeling.

**Interpretation:** Our results show that OCT imaging provides detailed insights into airway remodeling by reflecting extracellular matrix content changes, which correlates with asthma severity and poor control, complementing HRCT imaging’s measurement of airway wall thickness and offering potential for improved diagnostic and therapeutic strategies in asthma management.

Asthma, a chronic respiratory disease marked by recurrent episodes of wheezing, shortness of breath, cough, and chest tightness,^1^ is characterized by inflammation and structural changes in the airway wall. This structural transformation, known as *airway remodeling*, involves various alterations in the composition of the airway wall, including thickening of the smooth muscle layer, deposition of extra cellular matrix (ECM) proteins, and angiogenesis.^2^^,^^3^ Although these changes can lead to airway narrowing and bronchial hyperresponsiveness related to airflow obstruction, assessing airway remodeling remains a clinical challenge.^4^^,^^5^

Despite the significance of airway remodeling in asthma, assessing these structural changes remains a clinical challenge.^5^ Evaluation of specific airway wall components requires invasive biopsies, whereas the use of high-resolution CT (HRCT) imaging has emerged as a valuable technique to assess airway wall thickness (AWT).^6^, ^7^, ^8^, ^9^ This holds great potential in clinical settings, because bronchial wall thickening has been identified as one of the hallmark features distinguishing patients with asthma from healthy individuals.^10^, ^11^, ^12^ Although HRCT imaging has been proven useful, its resolution and poor soft tissue contrast preclude assessment of airway wall composition and specific airway disease hallmarks such as edema and inflammatory or structural changes.^13^ Therefore, interest in the application of optical coherence tomography (OCT) in pulmonary diseases is growing.^14^ OCT imaging is a high-resolution technique that uses the differences in light-scattering times from various tissue structures, allowing it to produce images with a resolution of ± 10 μm and an imaging depth of 2 to 3 mm.^15^ During bronchoscopy, an OCT imaging probe is inserted through the working channel of the bronchoscope, which enables real-time imaging from distal to proximal regions of the lung. Its high resolution enables imaging of airway wall composition.^16^, ^17^, ^18^, ^19^ Hence, OCT imaging holds potential for comprehensive and accurate assessment of airway remodeling in asthma in comparison with HRCT imaging, which can contribute to insight on the underlying mechanisms driving asthma pathogenesis. As such, minimally invasive OCT imaging of the airways is a promising alternative to focal, invasive biopsies, providing assessment of complete airway segments, rather than focal airway areas.

In a previous ex vivo study, we demonstrated the capability of OCT imaging to measure elastin and collagen content in the airway wall, reflecting ECM content.^20^ Although HRCT imaging allows measurement of the AWT, it does not provide information about ECM content. We hypothesized that bronchoscopic OCT imaging provides different and additional information than HRCT imaging by enabling assessment of ECM content. The aim of this study was to use both OCT and HRCT imaging to elucidate airway remodeling in asthma comprehensively. By focusing on the relationship between OCT imaging reflecting ECM components and asthma severity and parameters, we aimed to advance understanding of asthma pathogenesis and potentially to enhance diagnostic and therapeutic strategies.

## Study Design and Methods

### Study Design

This was a prospective study performed in the Amsterdam and Groningen University Medical Centers. We included healthy control participants, as well as patients with mild to moderate asthma and patients with severe asthma according to the Global Initiative for Asthma guidelines (GINA) (mild to moderate asthma, GINA steps 2-4; severe asthma, GINA step 5) who participated in the Asthma Origins and Remission (ARMS) study (Clinical trials.gov Identifier, NCT03141814) and Unravelling Targets of Therapy in Bronchial Thermoplasty in Severe Asthma (TASMA) study (Clinical trials.gov Identifier, NCT02225392). Ethical approval was obtained from the Medical Ethics Committee Amsterdam (Identifier, NL45394.018.13) and the Medical Ethics Committee Groningen (Identifier, NL53173.042.15). All participants gave their written informed consent.

### HRCT Imaging and Automated Analysis

Scans were obtained with a slice thickness of 0.75 or 1 mm and a pitch of 0.9 or 1.4 in a caudocranial scan direction to minimize breathing artefacts. The Yet Another CT Analyzer software versions 2.9.1.31 and 2.9.4.35 were used for fully automated airway analysis as described in earlier studies.^21^, ^22^, ^23^, ^24^, ^25^ Airway lumen and wall area were determined using the parameter-free integral-based method.^26^^,^^27^ A standardized measure for AWT was derived by plotting the square root of the airway wall area against the airway lumen perimeter for every measured airway location.

Then, by using the regression line of this plot, the square root of the wall area for a theoretical airway with an internal perimeter of 10 mm (Pi10) and an internal perimeter of 15 mm (Pi15) was determined, representing standardized AWT values for airways of different sizes.^21^, ^22^, ^23^, ^24^, ^25^^,^^28^ Analysis of airways with an internal perimeter of < 5 mm is prone to technical errors, and therefore was excluded.^9^

### In Vivo OCT Imaging and Segmentation

**Figure 1:**
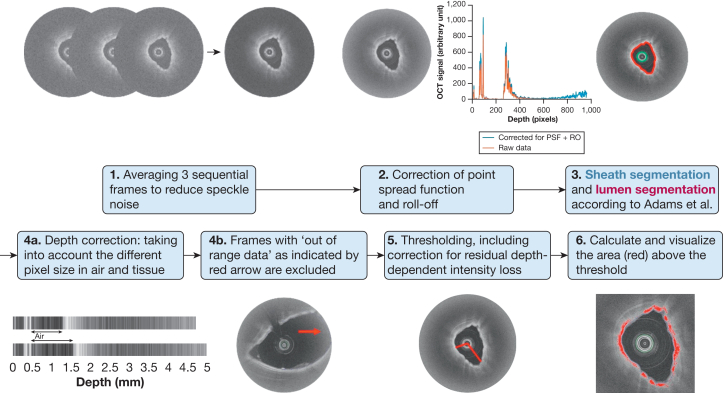
Diagram showing step-by-step analysis. In vivo OCT imaging high-intensity scattering area measurements were performed by an automated step-by-step analysis algorithm in cross-sectional OCT imaging airway wall images. OCT = optical coherence tomography; PSF = point ppread function; RO = readout noise.

During bronchoscopy, an OCT imaging probe (C7 Dragonfly; St. Jude Medical/Abbott) with an outer diameter of 0.9 mm was introduced through the working channel of the bronchoscope and positioned within the targeted airway. OCT imaging automated pullbacks of 5.4 cm from distal to proximal were performed in the right lower lobe and middle lobe. In each imaged airway, the pullback was performed twice. Stratified per 5 mm of OCT imaging pullback, high-quality cross-sectional airway images were selected for analysis. Fully automated OCT image segmentation for high-scattering intensity area analysis was performed with MATLAB (The MathWorks) in several steps (Fig 1). First, 3 sequential frames were averaged to reduce noise. Second, roll-off and point spread function corrections were performed to reduce system-related depth-dependent intensity loss, as described previously.^20^^,^^29^ Third, sheath and lumen segmentations were applied to distinguish tissue from air and probe.^30^ Fourth, these segmentations were used (1) to correct the image for the different pixel size in air (lumen) and tissue (airway wall) due to the difference in refractive index and (2) to exclude images in which part of the airway wall was outside the imaging range. Fifth, thresholding was performed with a correction for residual depth-dependent intensity loss. Finally, the resulting segmentation of high-intensity scattering was visualized and its area was calculated.

Data were grouped as in HRCT imaging data, based on OCT imaging-defined airway perimeter ranges: OCT imaging range of 2.5 to 7.5 mm as the internal perimeter of the < 5-mm group, OCT imaging range of 7.5 to 12.5 mm as the internal perimeter of the Pi10 group, and OCT imaging range of 12.5 to 17.5 mm as the internal perimeter of the Pi15 group. Every data point within each group was an average value per patient consisting of at least 2 measurements.

### Statistical Analysis

Baseline parameters were provided as mean (SD) for normally distributed values and as median (interquartile range) for nonnormally distributed values. Statistical comparisons between groups were conducted using appropriate tests, including the *t* test, Mann-Whitney *U* test, or χ^2^ test, depending on data characteristics such as normality and variable type. The differences in high-intensity scattering areas in the OCT images were evaluated using the 1-way analysis of variance with Bonferroni post hoc correction. Associations between patients’ clinical characteristics and OCT and HRCT imaging measurements were investigated using Pearson and Spearman correlation analysis, as appropriate based on data characteristics. Two-sided *P* values were used with a statistical significance at *P* < .05.

For each participant, we calculated the average OCT imaging high-intensity scattering area by analyzing data of multiple airway segments per participant, which included cross-sectional images categorized in internal perimeters of 2.5 to 7.5 mm, 7.5 to 12.5 mm, and 12.5 to 17.5 mm. R project version 4.4.0 software (R Core Team) and RStudio (Posit) version 2024.04.1 software were used for statistical computations.

## Results

### Participants

Forty-nine patients were included in this study, comprising 16 healthy control participants, 15 patients with mild to moderate asthma (GINA steps 2-4), and 18 patients with severe asthma (GINA step 5). Baseline demographic data, asthma control scores, and pulmonary function measurements are shown in Table 1.

**Table 1:** Baseline Characteristics of Participants

Variable	Control Group	Mild to Moderate Asthma Group	Severe Asthma Group
**No.**	16	15	18
**Sex, male**	10 (62.5%)^a^	11 (73.3%)^a^	3 (16.7%)^b^^,^^c^
Age, y	57.0 (53.3-59.5)^a^	56.0 (49.0-61.0)^a^	38.5 (29.3-52.0)^b^^,^^c^
**BMI, kg/m**^**2**^	24.6 (3.5)^a^	27.3 (6.2)	28.5 (5.1)^c^
Ethnicity			
Black	0 (0.0%)	0 (0.0%)	1 (5.6%)
Mixed	1 (6.7%)	0 (0.0%)	0 (0.0%)
White	14 (93.3%)	15 (100.0%)	17 (94.4%)
History of smoking	6 (37.5%)	2 (13.3%)	6 (33.3%)
ACQ-6 score	0 (0-0)	1.33 (0.4-1.4)^a^	2.67 (2.3-3.0)^b^
GINA treatment step			
2	0	3 (20.0%)	0 (0.0%)
3	0	7 (46.7%)	0 (0.0%)
4	0	5 (33.3%)	0 (0.0%)
5	0	0 (0.0%)	18 (100.0%)
Use of systemic corticosteroids	—	0 (0%)^a^	7 (38.0%)^b^
Use of biologicals	—	0 (0%)	2 (11.1%)
No. of exacerbations in the past year	—	0 (0-0)^a^	4.50 (1.3-9.0)^b^
**Allergy on skin prick test**	8 (50%)	11 (78.6%)	13 (72.2%)
**Blood**			
**Leukocytes, × 10**^**9**^**per L**	4.8 (4.5-5.8)^a^^,^^b^	6.2 (5.9-7.2)^c^	7.1 (5.6-9.4)^c^
**Eosinophils, × 10**^**9**^**per L**	0.1 (0.1-0.1)^b^	0.28 (0.2-0.4)^a^^,^^c^	0.15 (0.1-0.3)^b^
Neutrophils, × 10^9^ per L	2.9 (2.3-3.5)^a^^,^^b^	3.72 (3.2-4.3)^c^	4.14 (3.5-6.8)^c^
IgE	31.5 (22.0-62.0)^b^	119.5 (48.0-281.3)^c^	66.55 (17.1-169.5)
**Sputum**			
No. of obtained samples	7	8	5
Eosinophils, %	1.0 (0.4-1.0)	7.6 (0.9-13.2)	2.4 (0.2-3.0)
Macrophages, %	28.0 (27.5-41.5)^b^	20.2 (7.9-24.1)^c^	39.0 (12.0-50.0)
Lymphocytes, %	3.8 (2.0-4.4)	2.4 (1.7-3.7)	3.2 (2.1-3.8)
Neutrophils, %	58.0 (48.8-66.4)	71.3 (60.7-75.8)	53.0 (48.0-71.0)
**Spirometry**			
**FEV**_**1**_**, % predicted**			
**Before bronchodilator administration**	108.3 (12.2)^a^^,^^b^	79.8 (14.5)^c^	85.9 (19.8)^c^
**After bronchodilator administration**	112.2 (11.6)^a^^,^^b^	86.6 (13.1)^a^^,^^c^	97.3 (15.4)^b^^,^^c^
**FEV**_**1**_**reversibility, %**	2.9 (1.2-5.5)^a^^,^^b^	6.5 (4.3-13.9)^c^	11.1 (6.1-14.9)^c^
**PC20 methacholine**	32.0 (32.0-32.0)^a^^,^^b^	0.7 (0.4-2.3)^c^	0.5 (0.1-2.2)^c^
**FVC, % predicted**			
**Before bronchodilator administration**	111.9 (105.6-122.2)^a^^,^^b^	99.8 (91.9-106.9)	95.1 (81.8-101.9)^c^
**After bronchodilator administration**	144.3 (16.6)^a^^,^^b^	130.1 (15.8)^c^	120.3 (17.2)^c^
**FEV**_**1**_**to FVC ratio**			
**Before bronchodilator administration**	0.7 (0.7-0.8)^b^	0.6 (0.6-0.7)^a^^,^^c^	0.8 (0.7-0.8)^b^
**After bronchodilator administration**	0.8 (0.1)^b^	0.7 (0.1)^a^^,^^c^	0.8 (0.1)^b^
**Body plethysmography**			
**RV to TLC ratio before bronchodilator administration**	27.8 (26.6-33.5)^a^^,^^b^	35.4 (32.3-40.1)^a^^,^^c^	31.5 (25.0-33.0)^b^^,^^c^

Data are presented as No. (%), mean (SD), or median (interquartile range). ACQ-6 = 6-item Asthma Control Questionnaire; Bold = significant correlation with p <0.05; GINA = Global Initiative for Asthma; PC20 = methacholine provocation test; RV = residual volume; TLC = total lung capacity.

a *P* < .05 vs patients with severe asthma.

b *P* < .05 vs patients with mild to moderate asthma.

c *P* < .05 vs healthy control participants.

### HRCT Imaging Measurements

**Figure 2:**
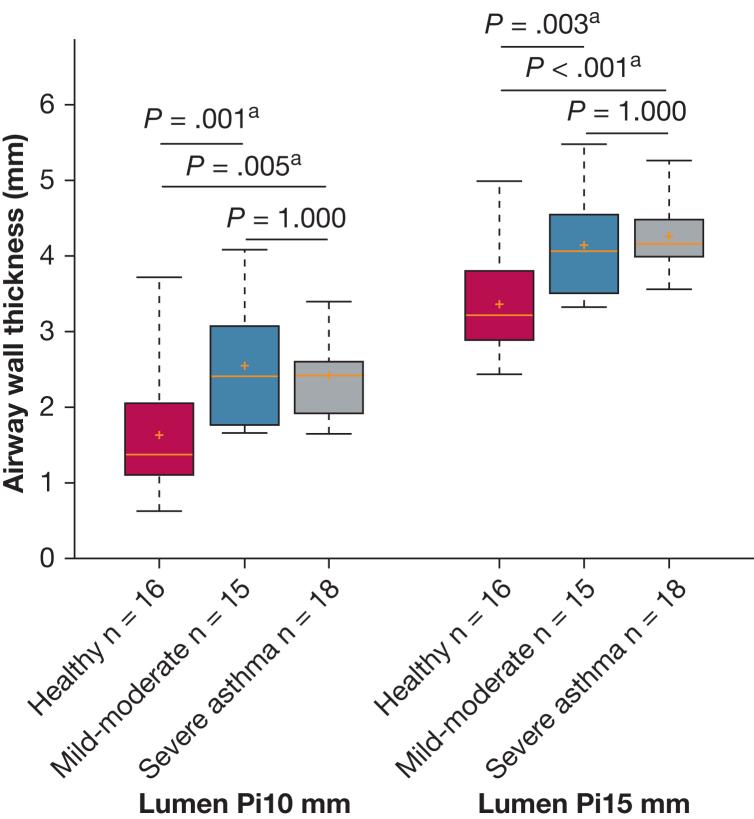
Box-and-whisker plots showing airway wall thickness measured by high-resolution CT imaging in Pi10 and Pi15. The whiskers represent the spread, the central line indicates the median, and the plus symbol indicates the mean. Pi15 = internal perimeter of 15 mm; Pi10 = internal perimeter of 10 mm.

The whole-lung AWT measured in Pi10 and Pi15 were higher for the mild to moderate group and severe group when compared with healthy control participants, but did not differ between patients with mild to moderate asthma and patients with severe asthma (Fig 2).

### OCT Imaging Measurements

**Figure 3:**
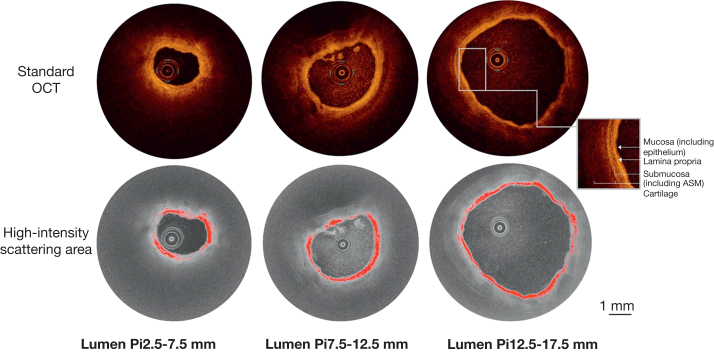
Cross-sectional OCT scans showing airway walls per lumen of Pi in a patient with severe asthma. The high-intensity scattering area, depicted by the red pixels, was measured by application of the algorithm (Fig 1) on the standard (original) OCT imaging cross-sections. ASM = airway smooth muscle; OCT = optical coherence tomography; Pi = internal perimeter.

**Figure 4:**
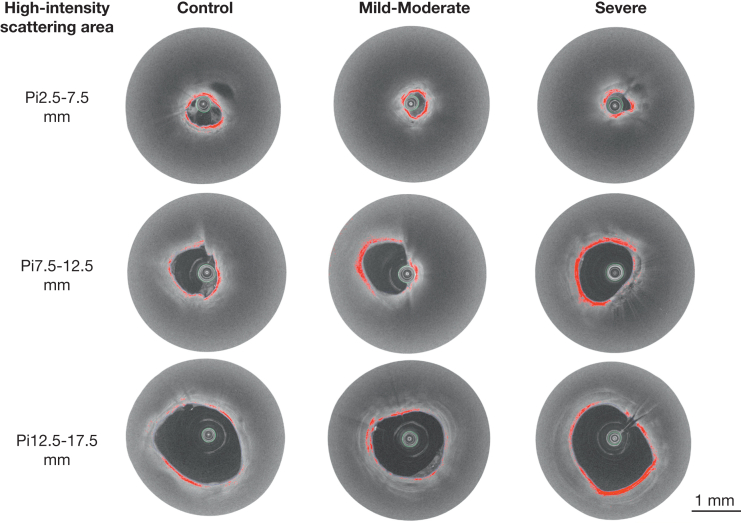
Cross-sectional optical coherence tomography (OCT) scans of airway walls per airway lumen Pi with the high-intensity area segmented in healthy control participants, patients with mild to moderate asthma, and patients with severe asthma. The high-intensity scattering area, depicted by the red pixels, was measured by application of the algorithm (Fig 1) on OCT imaging cross-sectional images. Pi = internal perimeter.

**Figure 5:**
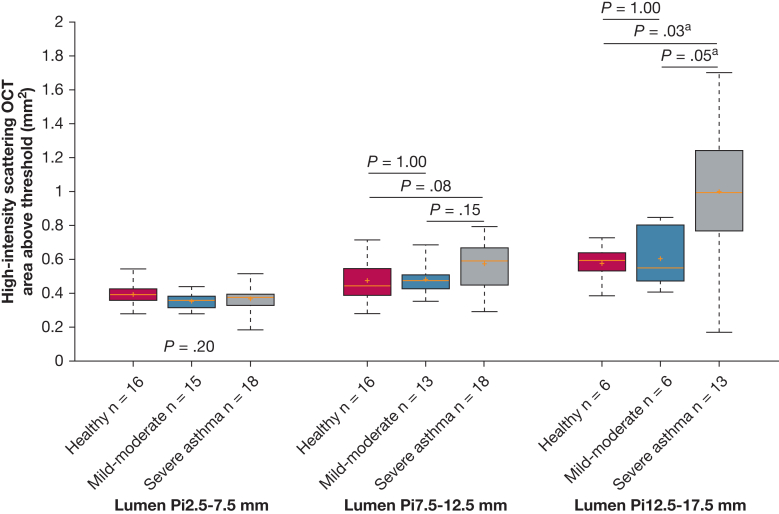
Box-and-whisker plots showing high-intensity scattering area measured by OCT imaging per lumen of Pi representing small, medium, and larger airways in healthy control participants, patients with mild to moderate asthma, and patients with severe asthma. OCT imaging high-intensity scattering area is for internal perimeters of 5 mm, 10 mm, and 15 mm. The whiskers represent the spread, the central line indicates the median, and the plus symbol highlights the mean. Pi = internal perimeter; Pi7.5-12.5 mm = airways with an internal perimeter between 7.5 and 12.5 mm; Pi12.5-17.5 mm = airways with an internal perimeter between 12.5 and 17.5 mm; Pi2.5-7.5 mm = airways with an internal perimeter between 2.5 and 7.5 mm.

In 49 patients, 1,763 OCT images were acquired over 130 airway segments. Representative scans of standard OCT imaging and overlay of high-intensity scattering area in cross-sectional images per lumen group are presented in Figure 3 and are categorized by internal lumen diameters, based on the categorization used for HRCT imaging. Figure 4 shows representative images with the area of high-intensity OCT imaging scattering from healthy control participants, patients with mild to moderate asthma, and patients with severe asthma per lumen of internal perimeter. The boxplots in Figure 5 represent all OCT imaging data showing the high-intensity scattering area. This area is increased toward the larger airways. In the airways with an internal perimeter between 12.5 and 17.5 mm, the OCT imaging high-intensity signal area is higher in patients with severe asthma when compared with the patients with mild to moderate asthma (*P* = .05) and healthy control participants (*P* = 0.03). In medium airways, a trend in increased high-intensity area in patients with severe asthma was detected as compared with the healthy control group (*P* = .08). The e-Figure 1 shows all the individual data points for each OCT imaging measurement per patient.

### Correlations With Clinical Characteristics

For all participants (healthy control participants, patients with mild to moderate asthma, and patients with severe asthma), we correlated both HRCT and OCT imaging-derived measurements with several clinical characteristics. The correlations of HRCT imaging with clinical characteristics are shown in Table 2. A thicker airway wall (HRCT imaging Pi10 and Pi15) was correlated with a lower FEV_1_ % predicted before and after bronchodilator administration. Additionally, a greater AWT correlated with an increase in the number of blood eosinophils and neutrophils, higher bronchodilator reversibility, asthma control scores (6-item Asthma Control Questionnaire [ACQ-6]), and bronchial hyperresponsiveness (methacholine provocation test [PC20]).

**Table 2:** Correlations Between HRCT Imaging-Derived Measures of Airway Wall Thickness and Clinical Characteristics of Participants, Grouped by Airway Lumen Size

Pi10			Pi15		
Variable	ρ/R	*P* Value	Variable	ρ/R	*P* Value
FEV_1_, % predicted			FEV_1_, % predicted		
Before bronchodilator administration	**–0.56**	**< .001**^a^	Before bronchodilator administration	**–0.58**	**< .001**^a^
After bronchodilator administration	**–0.56**	**< .001**^a^	After bronchodilator administration	**–0.57**	**< .001**^a^
FEV_1_ reversibility, %	**0.34**	**< .05**^a^	FEV_1_ reversibility	**0.34**	**< .05**^a^
FEV_1_ to FVC ratio	**–0.21**	**< .05**	FEV_1_ to FVC, ratio	–0.17	.24
RV to TLC ratio	0.21	.15	RV to TLC ratio	0.18	.21
PC20 methacholine	**–0.53**	**< .001**^a^	PC20 methacholine	**–0.50**	**< .01**^a^
Blood eosinophils, %	**0.47**	**< .01**^a^	Blood eosinophils, %	**0.41**	**< .01**^a^
Blood neutrophils, %	**0.36**	**< .05**^a^	Blood neutrophils, %	**0.42**	**< .05**^a^
Blood IgE	0.12	.40	Blood IgE	0.06	.67
Sputum eosinophils, %	0.11	.65	Sputum eosinophils, %	0.1	.69
Sputum lymphocytes, %	–0.11	.65	Sputum lymphocytes, %	–0.09	.70
Sputum macrophages, %	–0.13	.58	Sputum macrophages, %	–0.12	.61
ACQ-6	**0.45**	**< .01**^a^	ACQ-6	**0.55**	**< .001**^a^

ACQ-6 = 6-item Asthma Control Questionnaire; Bold = significant correlation with p <0.05; HRCT = high-resolution CT; PC20 = methacholine provocation test; Pi10 = internal perimeter of 10 mm; Pi15 = internal perimeter of 15 mm; RV = residual volume; TLC = total lung capacity.

a Significant correlation with *P* < .05.

For the OCT imaging measurements (Table 3), we found that in the smallest airways, the internal perimeter between 2.5 and 7.5 mm group, an increase of high-intensity scattering area correlated with a decrease in the number of blood and percentage sputum eosinophils, blood IgE and with less bronchial hyperresponsiveness (high-value PC20 results). In PC20 subanalysis with only patients with asthma (both mild to moderate and severe), the correlation between bronchial hyperresponsiveness and high-intensity scattering area remained present (*r* = 0.449; *P* = .015). In the internal perimeter between 12.5 and 17.5 mm group, we found a correlation between a decreased number of blood eosinophils and a larger OCT imaging high-intensity scattering area, in line with the internal perimeter between 2.5 and 7.5 mm group. A larger OCT imaging high-intensity scattering area also was associated with poorer asthma control scores (ACQ-6) within the internal perimeter between 7.5 and 12.5 mm and internal perimeter between 12.5 and 17.5 mm groups. No correlation was found between the HRCT imaging AWT and the OCT imaging high-intensity scattering area.

**Table 3:** Correlations Between OCT Imaging-Derived High-Intensity Scattering Area and Clinical Characteristics of Participants, Grouped by Airway Lumen Size

Pi2.5-7.5 mm			Pi7.5-12.5 mm			Pi12.5-17.5		
Variable	ρ/R	*P* Value	Variable	ρ/R	*P* Value	Variable	ρ/R	*P* Value
FEV_1_, % predicted			FEV_1_, % predicted			FEV_1_, % predicted		
Before bronchodilator administration	0.25	.09	Before bronchodilator administration	0.01	.93	Before bronchodilator administration	–0.16	.44
After bronchodilator administration	0.18	.22	After bronchodilator administration	–0.01	.93	After bronchodilator administration	0.002	.99
FEV_1_ reversibility, %	–0.26	.08	FEV_1_ reversibility	0.13	.38	FEV_1_ reversibility	0.16	.43
FEV_1_ to FVC, ratio	0.16	.29	FEV_1_ to FVC, ratio	0.24	.11	FEV_1_ to FVC, ratio	0.28	.18
RV to TLC ratio	0.10	.48	RV to TLC ratio	0.14	.35	RV to TLC ratio	0.03	.91
PC20 methacholine	**0.31**	**< .05**^a^	PC20 methacholine	-0.08	.63	PC20 methacholine	–0.06	.79
Blood eosinophils	**–0.37**	**< .01**^a^	Blood eosinophils, %	–0.22	.15	Blood eosinophils	–**0.42**	**< .05**^a^
Blood neutrophils	–0.03	.83	Blood neutrophils, %	**0.34**	**< .05**^a^	Blood neutrophils	0.33	.11
Blood IgE	**–0.44**	**< .01**^a^	Blood IgE	–0.23	.13	Blood IgE	–0.29	.17
Sputum eosinophils, %	**–0.60**	**< .01**^a^	Sputum eosinophils, %	–0.26	.28	Sputum eosinophils, %	–0.21	.59
Sputum lymphocytes, %	–0.17	.47	Sputum lymphocytes, %	0.07	.76	Sputum lymphocytes, %	–0.19	.62
Sputum macrophages, %	0.21	.37	Sputum macrophages, %	0.17	.49	Sputum macrophages, %	0.08	.83
ACQ-6	–0.16	.28	ACQ-6	**0.32**	**< .05**^a^	ACQ-6	**0.60**	**< .01**^a^

ACQ-6 = 6-item Asthma Control Questionnaire; Bold = significant correlation with p <0.05; OCT = optical coherence tomography; PC20 = methacholine provocation test; Pi7.5-12.5 mm = internal perimeter of 7.5 to 12.5 mm; Pi12.5-17.5 mm = internal perimeter of 12.5 to 17.5 mm; Pi2.5-7.5 mm = internal perimeter of 2.5 to 7.5 mm; RV = residual volume; TLC = total lung capacity.

a Significant correlation with *P* < .05.

## Discussion

To our knowledge, this study is the first to use automated OCT imaging analyses for in vivo assessment of airway extracellular matrix components in patients with asthma and healthy control participants. We showed that both OCT and HRCT imaging provide different insights into airway remodeling. The OCT imaging high-intensity scattering area, reflecting an increased elastin and collagen deposition in the airway wall as demonstrated in earlier work,^20^ was found to be increased in the group with severe asthma. This finding is in line with previous studies, reflecting that airway remodeling is associated with asthma progression.^31^^,^^32^ AWT, measured by HRCT imaging, showed differentiation between healthy and asthmatic airways. However, it was unable to discriminate between the varying degrees of asthma severity. Our findings highlight the complementary aspects of these imaging methods and collectively provide novel insights on our understanding of airway remodeling in asthma.

No correlation was found between the elastin and collagen content measured by OCT imaging and AWT measured by HRCT imaging. This finding implies that collagen and elastin content are not per se increased in individuals with a thicker airway wall. Alternatively, edema, inflammatory cells, and other residential cells, like epithelial and smooth muscle cells, may lead to an increase in airway wall thickness,^33^^,^^34^ reflecting that airway remodeling is a complex process. Interestingly, less asthma control (reflected by a higher ACQ-6 score) correlated with both AWT and high-intensity scattering area, which indicates that both parameters serve as markers for poor asthma control and asthma severity.^35^^,^^36^

When assessing different clinical characteristics for asthma, our results confirm that an increase in AWT correlated with a decrease in FEV_1_ and an increase in airway reversibility in patients with asthma and healthy control participants, as shown in previous studies.^9^^,^^37^ This reinforces the idea that an increased AWT contributes to airflow obstruction. Unlike HRCT imaging, we did not find a direct correlation between the OCT imaging high-intensity scattering area and airflow obstruction (FEV_1_), although, we did find a negative correlation between bronchial hyperresponsiveness and OCT imaging high-intensity scattering area in the smallest airways, indicating that collagen and elastin content may have a stabilizing effect on bronchial hyperresponsiveness.

Considering the different groups, we found that FEV_1_ and FEV_1_ to FVC ratio demonstrated a greater degree of impairment within the group with mild to moderate asthma in comparison with the group with severe asthma. However, FEV_1_ reversibility and ACQ-6 score were worse in the severe group, and participants in the severe group experienced more exacerbations, supporting current guidelines stating that the definition of severe asthma is not solely based on FEV_1_.^1^ Next to lung function, the eosinophil blood count was higher in the patients with mild to moderate asthma compared with the patients with severe asthma. The lower eosinophil count in the patients with severe asthma is possibly related to the use of high-dose inhaled and oral corticosteroids. No differences were found in the percentage of sputum eosinophils, but the number of obtained sputum samples was low, which could have affected the power of this analysis. Although oral and inhaled corticosteroid use was more prevalent in patients with severe asthma, we did observe a link between HRCT imaging wall thickness and an increase in eosinophilic inflammation. With OCT imaging, a negative association was found between high-intensity scattering area and eosinophilic inflammation, suggesting involvement of alternative mechanisms on collagen and elastin changes. This is in line with previous research in which the effect of eosinophilic inflammation on extracellular matrix components changes is not understood completely,^38^ although eosinophilic-derived proteins have been described as influencing airway remodeling.^39^ A better understanding of the factors driving airway remodeling is crucial. Considering the ability to measure components of airway remodeling directly by OCT imaging and its ability to mark distinctive features of severe asthma, we suggest that further research should focus on the use of OCT imaging in combination with techniques like RNA sequencing and proteomics for comprehensive analyses of airway remodeling and how this relates to various inflammatory processes within the airway wall.

Increased high-intensity OCT imaging signal, which reflects ECM components such as collagen and elastin content, was observed in the medium airways (internal perimeter of 12.5-17.5 mm) of patients with severe asthma when compared with patients with mild to moderate asthma and healthy participants, but not in the smaller airways (internal perimeter < 7.5 mm). This implies that the change in these structural components of the airway wall seems particularly prominent in the medium airways and challenges the hypothesis of asthma being solely a small airways disease.^40^ Instead, it suggests that changes in the extracellular matrix occur mainly in the airways with an internal perimeter between 12.5 and 17.5 mm. Histologic studies have described changes in collagen and elastin content in the asthmatic airway wall,^4^ with mixed results for elastin,^41^ and mainly have investigated different subtypes of collagen.^4^^,^^42^ However, biopsy examinations offer limited assessment of the airways because they target focal small airways wall areas. Furthermore, HRCT imaging lacks the resolution necessary to provide insight into the smallest airways and airway wall composition. In contrast, OCT imaging is able to visualize complete airway segments with various sizes, including the small airways, thereby offering new insights. Our data suggest that a difference exists in high-intensity OCT imaging signal in the internal perimeter of 2.5 to 7.5 mm group compared with the larger airways. This is in line with histologic studies demonstrating that the extent of airway remodeling can vary between the larger and smaller airways.^43^

One of the outcomes of this study suggests that higher elastin and collagen content corresponds with less bronchial hyperresponsiveness. Because bronchial hyperresponsiveness is not present in healthy participants, a subanalysis was performed within the groups with asthma, where we also found a relationship between the high-intensity scattering area and less bronchial hyperresponsiveness. This is in contrast with an earlier study, where an increase of elastin was correlated with more bronchial hyperreactivity, although this study measured elastin content only outside the airway smooth muscle bundle as opposed to full airway wall measurements.^44^ However, dividing the PC20 measurements within various airway sizes should be performed with caution because the responsiveness in bronchoprovocation tests may vary between larger and smaller airways.^45^^,^^46^

One of the strengths of this study is the inclusion of a healthy control group, which provides a valuable reference for comparing both the HRCT and OCT imaging measurements in patients with asthma. This enables the ability to discern the structural changes associated with asthma from the background variations that might occur in patients without asthma. Further, we were able to provide insight into airway remodeling of the small airways by the use of OCT imaging, which is not feasible with HRCT imaging. Finally, the inclusion of patients with both mild and severe asthma provides further insight into structural alteration within the airway wall across various levels of asthma severity. Some limitations have to be acknowledged as well. First, asthma is a heterogeneous disease, and the relatively small size of the study cohort may limit the generalizability of the findings in the population with asthma including different phenotypes. Second, we found a disbalance between the groups with respect to age: patients with mild to moderate asthma on average were older than those with severe asthma. Importantly, all patients with mild to moderate asthma experienced early disease onset and therefore a longer duration of asthma, with an extended time for remodeling to develop. Nevertheless, we still found a higher degree of high-intensity signal area indicating airway remodeling with ECM deposition in patients with severe asthma. Third, we did not analyze AWT or airway smooth muscle with OCT imaging, because the preferred method to analyze airway smooth muscle is polarization-sensitive OCT imaging,^47^ which we did not apply in the current study. Measurement of AWT was unavailable because the automatic segmentation method used in this analysis did not encompass this function. Finally, the classification of the OCT imaging groups into various lumen perimeters was determined following the previously used HRCT imaging categorization.^21^^,^^28^^,^^48^ As a consequence, the number of acquired images is smaller in the group with 12.5- to 17.5-mm OCT imaging.

## Interpretation

This study presents a comprehensive comparison between OCT and HRCT imaging findings in healthy participants and patients with asthma. Although HRCT imaging is valuable in assessing airway wall thickness, it falls short in providing detailed information of airway wall composition. OCT imaging is a high-resolution imaging technique that reflects ECM content, including collagen and elastin, which increases with asthma severity and correlates with poor asthma control. Incorporating both HRCT and OCT imaging techniques in future research can advance our understanding of asthma’s structural airway wall changes significantly and potentially can lead to more targeted and personalized therapeutic approaches.

## Funding/Support

This study was supported by the Netherlands Lung Foundation [Grants: 5.2.13.064JO and 5.1.14.020], the European Union’s H2020 Research and Innovation Program [Grant 874656 (discovAIR)], 10.13039/501100004344Stichting Astma Bestrijding [Grant 1018/041], The Netherlands Organization for Health Research and Development [Grant: 90713477], and 10.13039/100008497Boston Scientific Corporation. This publication is part of the project 2 “Structural and Florescence Imaging in Lung Cancer and Asthma” of the research program Medphot, which is financed in part by the Dutch Research Council. M. C. N. is supported by grants to his institution from European Union’s H2020 Research and Innovation Program under grant agreement, the Ministry of Economic Affairs and Climate Policy (The Netherlands) through a PPP allowance from the Top Sector Life Sciences & Health, GSK Ltd. Stevenage (UK), Netherlands Lung Foundation, The Chan Zuckerberg Initiative, and The Stichting Astmabestrijding. C. J. G. is supported by the 10.13039/100000050National Heart, Lung, and Blood Institute, National Institutes of Health [Grant R01HL139690].

## Financial/Nonfinancial Disclosures

The authors have reported to *CHEST Pulmonary* the following: M. C. N. support of travel costs by the Belgian Respiratory Society and unpaid leadership of the Lung Bionetwork of the Human Cell Atlas consortium. C. J. G. reports a financial interest in Imbio, LLC, and is supported by the National Heart, Lung, and Blood Institute, National Institutes of Health. O. W. reports licences to Imbio, LLC. M. v. d. B. reports grants paid to his university from GSK, Chiesi, Teva, Astra-Zeneca, and Genentech, outside the submitted work. None declared (P. C. W., L. H. v. S., R. M. v. d. E., A. W. M. G., J. N. S. d’H., O. A C., P. R. B., I. A. H. v. d. B., A. J. B., D. M. d. B., J. T. A., P. I. B.).

## Figures and Tables

**e-Figure:**
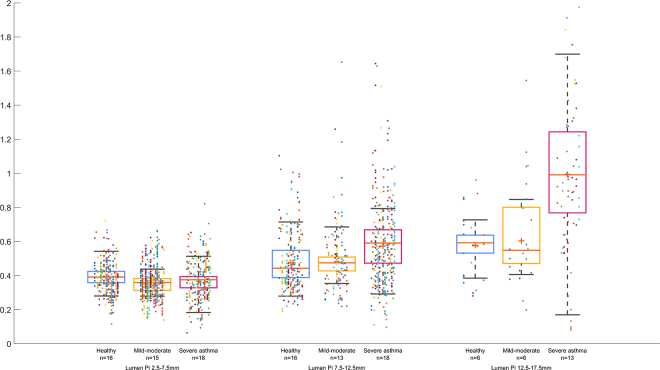


## Supplementary Data

e-Online Data

e-Online Data
