# Supplementary material for: Detection of Airway Remodeling in Asthma Using Bronchoscopic Optical Coherence Tomography
Source: CHEST Pulm. 2025 Feb 24;3(3):100143. doi: 10.1016/j.chpulm.2025.100143 (PMC13418684; doi:10.1016/j.chpulm.2025.100143)
Supplement: e-Online Data [file mmc2.docx]

**Data supplement**

*Supplementary Figure 1.* Boxplots representing high intensity scattering area measured by OCT in healthy control subjects, mild-moderate and severe asthma patients per Lumen Pi representing small, medium and larger-sized airways.

**
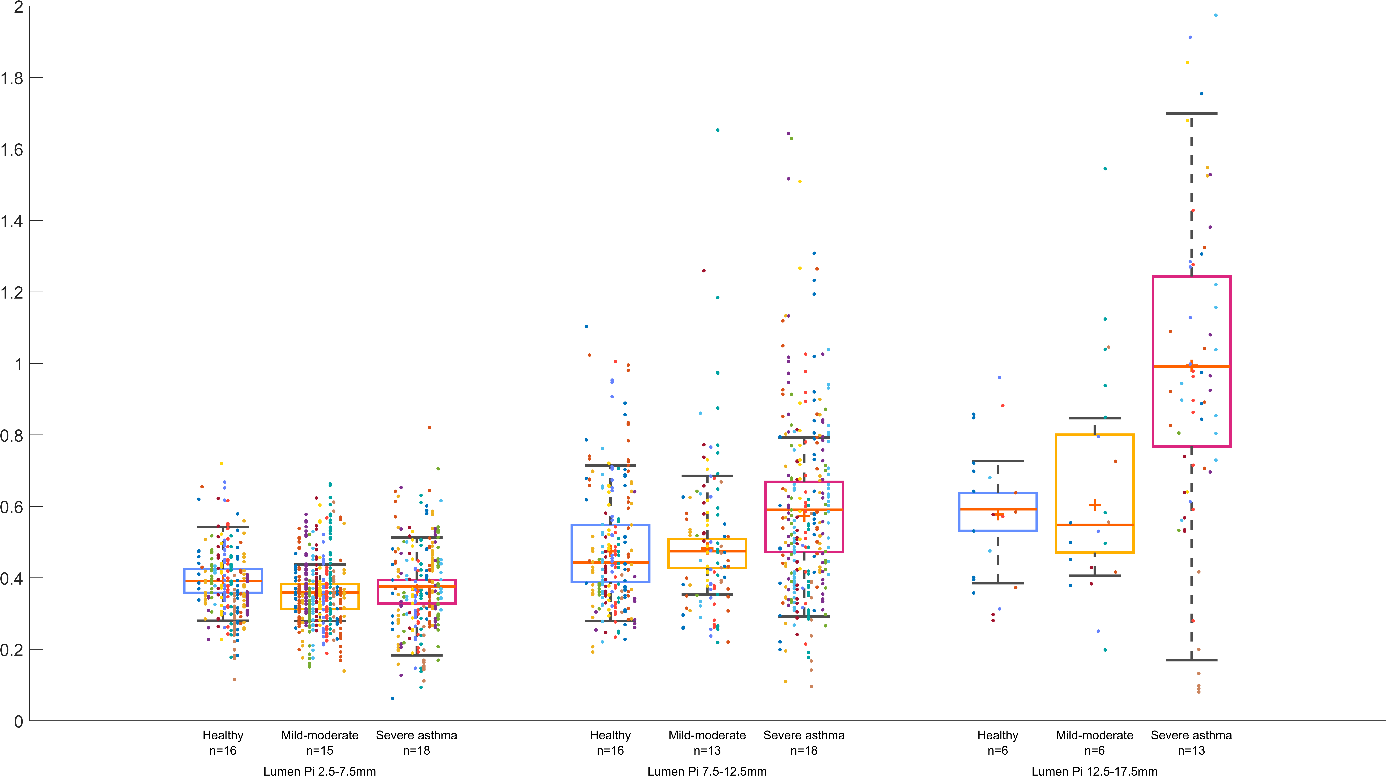
**

*Supplementary Figure 1*. Each dot represents an individual data point for each OCT measurement per patient, with different colors representing different individuals. The whiskers represent the spread, while the central line indicates the median, and the "+" symbol highlights the mean.
